# The Value of Quantitative Ultrasound Elastography in the Assessment of Non-Alcoholic Fatty Liver Disease in Children

**DOI:** 10.2174/0115734056373132250507141342

**Published:** 2025-05-15

**Authors:** Xu Cao, Jianbo Liu, Jing Li, Kexin Shi, Shuang Zheng, Dongna Di, Peng Tian

**Affiliations:** 1 Ultrasound Medicine, Mudanjiang Medical University, Mudanjiang 157011, Heilongjiang, China; 2 Department of Ultrasound Medicine, The Sixth Affiliated Hospital of Mudanjiang Medical University, Daqing Oilfield General Hospital, Daqing 163001, Heilongjiang, China

**Keywords:** Sound touch elastography, Sound touch quantification, Children, Fatty liver disease, Shear wave elastography, Ultrasound

## Abstract

**Objective:**

This preliminary investigation aimed to assess the value of two elastography techniques, sound touch elastography (STE) and sound touch quantification (STQ), in measuring liver stiffness in children with non-fatty versus fatty livers.

**Methods:**

This study used a case-control design. The STE and STQ were used to measure and compare liver stiffness in 121 children with fatty livers and 251 children with non-fatty livers, respectively.

**Results:**

In this study, we found that, compared to children with non-fatty liver disease, children with fatty liver disease had lower Young's modulus values in STE and STQ in the left lobe of the liver, and the difference was statistically significant (*P* < 0.05). However, after multifactorial analysis, no association was found between liver Young's modulus values measured by STE and STQ and the presence of fatty liver in children.

In the present study, significantly higher Young's modulus values were observed in the left lobe compared to the right lobe of the liver in children with non-fatty liver (*P* < 0.05). In contrast, no significant difference was found between the left and right lobes in children with fatty liver (*P* > 0.05). The optimal diagnostic threshold for detecting steatohepatitis in the left lobe was 5.890 kPa using STE and 8.050 kPa using STQ.

**Conclusion:**

STE and STQ, as the latest ultrasound diagnostic techniques based on shear wave elastography, can quantitatively assess fatty liver in children. In this study, some liver elasticity measurements in the fatty liver group differed from those in the non-fatty liver group.

## 1 INTRODUCTION

Non-alcoholic fatty liver disease (NAFLD) is characterized by the abnormal accumulation of fat in liver cells due to non-alcoholic factors and is currently the leading chronic liver disease in the world [1]. NAFLD disease progression consists of two main stages: Non-alcoholic fatty liver (NAFL) and Non-alcoholic steatohepatitis (NASH). NAFL is defined by the presence of steatosis in ≥5% of hepatocytes without evidence of significant intralobular inflammation or fibrosis. In contrast, NASH is characterized by ≥5% steatosis accompanied by hepatocellular injury and inflammation, including portal inflammation [2]. Obesity is closely associated with an increase in the prevalence and severity of NAFLD [3]. In recent years, with the development of the Chinese economy and the improvement of living standards, overweight and obese children are also increasing. Although NAFLD is not an acute and serious condition, prolonged NAFL can progress to NASH, which ultimately leads to abnormal liver function, cirrhosis, and end-stage liver disease. Therefore, its early diagnosis and prevention is the key to preventing its progression. So far, the gold standard for evaluating NAFLD is liver biopsy, but the test is invasive and not well accepted by children themselves and their parents. CT, MRI, and 2D ultrasound imaging are commonly used in the clinical diagnosis of fatty liver. However, CT involves radiation exposure, has limited reproducibility, and does not permit real-time dynamic assessment of the liver. MRI, while highly accurate, is costly and time-consuming, making it less practical for routine or large-scale screening. The limitations of 2D ultrasound are that the diagnosis is highly subjective and can only be assessed qualitatively. In recent years, with the development of ultrasound medical technology and the application of new techniques, ultrasound elastography (UE), as a new ultrasound diagnostic technique, can non-invasively and quantitatively assess liver stiffness. Sound touch elastography (STE) and sound touch quantification (STQ) are the latest techniques based on shear wave elastography (SWE). The ultrasound probe emits focused acoustic radiation force pulses at varying tissue depths, generating a force that induces a transverse shear wave. Ultra-high-speed imaging technology captures this shear wave, producing a two-dimensional shear wave image. From this, the Young's modulus (kPa) of the liver can be calculated, providing a measure of the liver's elasticity and hardness [4]. STE is a form of two-dimensional shear wave elastography (2D-SWE), while STQ is classified as point shear wave elastography (pSWE). Both are based on 2D-SWE with real-time imaging to detect liver stiffness and have wide applicability because they are not affected by obesity, ascites, or narrowing of the intercostal space [5]. Several studies have reported the good diagnostic performance of 2D-SWE for diagnosing various stages of fibrosis in NAFLD patients [6, 7], but using SWE to assess liver stiffness in pediatric NAFLD is rarely reported. This study aimed to investigate the application of two SWE models, STE and STQ, respectively, to assess liver stiffness in normal and NAFLD children in an attempt to explore the quantitative detection and diagnosis of NAFLD.

## 2 METHODS AND MATERIALS

### 2.1 Study Participants

A total of 372 children hospitalized in our pediatric department from March, 2023 to January, 2024 were randomly selected for the study. Two-dimensional ultrasound confirmed the diagnosis of fatty liver in 121 cases (fatty liver group). Among them, 88 were males, and 33 were females. Moreover, 251 cases were categorized in the non-fatty liver group by two-dimensional ultrasound (control group), comprising 129 males and 122 females. This study was approved by the Ethics Committee of Daqing Oilfield General Hospital (Approval No.: ZYAF/SC-07/02.0). Written informed consent was obtained from all participants prior to their inclusion in the study. The study objectives, procedures, potential risks, and benefits were thoroughly explained to the participants. Participants were explicitly informed of their right to withdraw from the study at any time without affecting their routine medical care. For minor participants, informed consent was additionally obtained from their legal guardians.

#### 2.1.1 Inclusion Criteria

The inclusion criteria are as follows:

(1) Children under 13 years of age.

(2) The examined children were subjected to STE and STQ measurements and two-dimensional ultrasound.

(3) Patients who were able to cooperate in completing the ultrasound examination.

(4) Adequate communication with and consent from parents prior to the examination.

#### 2.1.2 Exclusion Criteria

The exclusion criteria are as follows:

(1) Children with specific liver diseases, such as viral hepatitis, drug-induced liver disease, and autoimmune liver disease.

(2) Inability to perform respiratory fit, STE, and STQ testing failed to yield satisfactory results with prescribed quality control.

### 2.2 Instruments and Methods

#### 2.2.1 Instruments and Equipment

The Mindray Reaona R9 color Doppler ultrasound diagnostic instrument, equipped with an SC6-IU type convex array probe operating at a frequency of 3–5 MHz, was used in this study.

#### 2.2.2 Participants Preparation

The children were examined early in the morning on an empty stomach, in a lying position with the hands raised above the head for the examination of the left lobe of the liver, and in a left lateral position with the right hand placed above the head for the measurement of the right lobe of the liver, exposing the right quaternary ribcage area, and all the measurements were taken during pauses for respiration.

#### 2.2.3 General Information

The gender, age (years), height (m), and weight (kg) of the enrolled patients were recorded, and their body surface area (m^2^) and BMI (kg/m^2^) were calculated according to the formula.

#### 2.2.4 2D Ultrasound of the Liver

Using the SC6-IU convex array probe with a frequency range of 3–5 MHz, the liver parenchyma, intrahepatic blood vessels, and bile ducts were observed to assess pediatric abdominal conditions. Color Doppler imaging was used to evaluate portal blood flow, and for obese children, the depth of the image could be adjusted by rotating the depth button to obtain high-quality images.

#### 2.2.5 Two-Dimensional Ultrasound for the Diagnosis of Fatty Liver [8]

The criteria for diagnosing fatty liver using two-dimensional ultrasound are:

(1) The liver appears enlarged, with rounded margins at the corners.

(2) Enhanced near-field echogenicity of the liver parenchyma, with stronger echogenicity than that of the kidneys and spleen.

(3) Poor visualization of the liver’s internal ductal structure.

(4) Attenuation of the far-field echo signal, with a poorly defined liver contour.

The EASL-EASD-EASO Clinical Practice Guidelines for the management of non-alcoholic fatty liver disease [9] recommend two-dimensional ultrasound (2D-US) as the preferred imaging modality for hepatic steatosis detection, owing to its wider availability and lower cost compared to MRI. Despite operator dependence, 2D-US demonstrates reliable diagnostic accuracy for moderate-to-severe hepatic steatosis, with a sensitivity of 85% (95% CI: 80%-89%) and specificity of 93% (95% CI: 87%-97%), as supported by a large-scale meta-analysis using liver biopsy as the reference standard. Additionally, its ability to provide supplementary hepatobiliary information reinforces its role as a dependable first-line imaging tool for NAFLD risk assessment in clinical practice. Therefore, this study adopts 2D-US as the diagnostic standard for NAFLD [10].

#### 2.2.6 STE and STQ Measurement

Shear wave elastography was conducted using the Resona R9 ultrasound diagnostic instrument, with the SC6-1U convex array probe selected, operating at a frequency of 3–5 MHz:

(1) The probe was placed in a long-axis view of the abdominal aorta just below the xiphoid process, providing a longitudinal view of the left outer lobe of the liver while avoiding the intrahepatic bile ducts and large blood vessels. This allowed for a clear display of the liver parenchyma in 2D. The STE mode was activated with a sampling frame size of 4 cm × 3 cm, and the region of interest (ROI) was set to 1 cm × 1.5 cm, located approximately 1–2 cm from the liver peritoneum, with a maximum depth of 8 cm [11]. The participants were instructed to hold their breath (2 to 4 s). For participants who could not or were too young to hold their breath, this was done during quiet breathing, and when the image stabilized, the update button was pressed after the Resona 9 system provided reliability (RLB) metrics and RLB plots for the motor stability (M-STB) metrics. The criteria for checking reliability include [12]:1. M-STB index, consisting of 5 stars; results are reliable when 4 or more stars are displayed in green; 2. RLB map: the color consists of purple-green; when the color is mostly green, the result is reliable; 3. RLB index: expressed by the percentage, when more than 90% of the results are reliable, the liver stiffness measurement (LSM) is considered valid. The freezing key was pressed, and the region of interest (ROI) diameter was set to 15 mm. STE measurements were performed on the left lobe of the liver, with three measurements taken and averaged. The participants were then instructed to relax, and the liver parenchyma was clearly visualized in 2D. Afterward, the STQ mode was initiated, operating as in STE above. When the M-STB index showed at least four stars and IQR/MED ≤ 30%, LSM was considered valid. The freeze button was pressed, the ROI region of interest was adjusted to 1.5cm×1.0cm, and the STQ measurement of the left lobe of the liver was carried out. The measurement was repeated three times to take the average value.

(2) Participants were instructed to lie on their left side and raise their right arm above their head to fully expose the intercostal space. By avoiding large blood vessels and the gallbladder, the probe was then placed in the right intercostal oblique section (right anterior lobe of the liver) [13], and the same procedure was followed as described above to measure the STE and STQ ultrasound elastography values.

### 2.3 Sample Size Calculation

This study calculated the required sample size based on expected left lobe liver stiffness (LSM) differences. Preliminary data showed LSM values of 8.84 kPa (control) and 7.01 kPa (experimental), with a standard deviation of 2.15 kPa. Using PASS 15.0 for a two-sample t-test (α=0.05, power=0.80), the initial sample size was 27 (experimental) and 54 (control). Accounting for 20% attrition, the final target was 32 and 64, respectively.

During implementation, 372 patients were enrolled (121 experimental and 251 control), exceeding requirements. However, despite the large sample, right lobe LSM differences may lack statistical significance, possibly due to anatomical or technical factors. Future studies should use a larger sample size and refine measurement methods for more robust results.

### 2.4 Statistical Methods

Data were analyzed and counted using SPSS statistical software. Measurements obeying normal distribution are expressed as mean ± standard deviation. Independent samples t-tests were carried out to compare between-group differences for continuous variables, and chi-square tests were conducted to compare between-group differences for categorical variables. The association of right lobe STE, left lobe STE, right lobe STQ, and left lobe STQ with NAFLD was explored using binary logistic regression. To construct the receiver operating characteristic curve (ROC), the area under the curve (AUC) was calculated, and the ability of right lobe STE, left lobe STE, right lobe STQ, and left lobe STQ to diagnose NAFLD was analyzed. *P* < 0.05 indicated that the difference was statistically significant.

## 3 RESULTS

### 3.1 Baseline Characterization

Three hundred seventy-two participants were included. Among them, 217 were males, and 155 were females, which were further categorized into 121 children with fatty liver and 251 children with normal liver. As mentioned in Table **1**, study participants in the fatty liver group had significantly greater age, height, weight, BMI, and body surface area compared to those in the non-fatty liver group (*P* < 0.01). Additionally, the fatty liver group consisted predominantly of male participants (*P* < 0.01).

### 3.2 Comparison of Mean Young's Modulus Values between children with non-fatty and fatty livers

The fatty liver group showed significantly lower Young’s modulus values in the left lobe of the liver, both in STE (7.31 ± 2.09 kPa *vs*. 5.47 ± 1.06 kPa, *P* < 0.001) and STQ measurements (8.84 ± 3.26 kPa *vs*. 7.01 ± 2.15 kPa, *P* < 0.001), compared to the non-fatty liver group. The difference in STE and STQ of the right lobe of the liver in children was not statistically significant between the non-fatty and fatty liver groups (*P* > 0.05).

### 3.3 Comparison of Mean Young's Modulus Values of the Left and Right Lobes of the Liver

In the non-fatty liver group, the left lobe showed significantly higher STE-derived Young’s modulus values compared to the right lobe (7.31 ± 2.09 kPa *vs*. 6.33 ± 1.42 kPa, *P* < 0.001). In contrast, in the fatty liver group, no statistically significant difference was observed between the left and right lobes in either STE or STQ measurements (*P* > 0.05).

### 3.4 Multivariate Logistic Regression Analysis of Fatty Liver Disease

Several variables were included in the univariate regression analysis, and then the multivariate logistic regression analysis was conducted. The results indicated that BMI and the STE-derived Young’s modulus value in the left lobe of the liver were independent risk factors for NAFLD. BMI showed a significant positive association with the presence of fatty liver, with an OR value of 2.61, indicating that each one-unit increase in BMI was associated with a 2.61-fold higher risk of NAFLD. In contrast, liver stiffness measured by SWE in the left lobe demonstrated a significant negative association with NAFLD, with an OR value of 0.61, suggesting that for each one-unit increase in liver stiffness, the risk of NAFLD decreased by 0.61 times.

### 3.5 ROC Curve Analysis of STE and STQ in the Right and Left Lobes of the Liver

The ROC curve was plotted using the STE-derived Young's modulus value of the left lobe of the liver to differentiate fatty liver from non-fatty liver, and the area under the curve was 0.794. The optimal diagnostic cut-off value was 5.890 kPa, and a diagnosis of fatty liver was made at a value of less than 5.890 kPa, with a diagnostic sensitivity of 74.8% and a specificity of 75.2%. The ROC curve was plotted using the STQ-derived Young's modulus value of the left lobe of the liver to differentiate fatty liver from non-fatty liver, with an area under the curve of 0.688 and the optimal diagnostic cut-off value of 8.050 kPa. A diagnosis of fatty liver was made at a value of less than 8.050 kPa with a diagnostic sensitivity of 51.6% and a specificity of 77.7%. The ROC curve for the diagnosis of non-alcoholic fatty liver using liver Young's modulus values is shown in Fig. (**1**).

## 4 DISCUSSION

In the present study, data from 372 hospitalized children were analyzed, and it was found that the fatty liver group had lower values of Young's modulus of the left lobe of the liver than the non-fatty liver group, and the difference was statistically significant (Table **2**). This may be due to an excessive accumulation of fat in the liver cells. It has been observed that this fat usually accumulates in the cytoplasm of hepatocytes in the form of triacylglycerols, forming small droplets of fat. Microscopically, vacuoles of varying sizes appear in the cytoplasm of the degenerated hepatocytes, increasing the intercellular space and thus decreasing the density of the hepatic tissue, resulting in a softer texture of the liver compared to that of normal liver tissue, which leads to a lower value of liver stiffness than that of normal liver tissue as assessed by elastography [14]. Wang *et al.* [15] compared hepatic Young’s modulus values between patients with fatty liver disease and a healthy control group and found that the fatty liver group exhibited lower values than the normal population, a result consistent with the findings of the present study. However, there are also studies demonstrating contradictory results to the present study. Garcovich *et al.* applied SWE and found a significant positive correlation between LSM and the degree of liver fibrosis in children with NAFLD [16]. Bailey *et al.* applied SWE and found that the mean SWE velocity measurements were significantly higher in the obese group (1.44 ± 0.39 m/s) compared to the normal group (1.08 ± 0.14 m/s), suggesting more severe hepatic fibrosis in obese children (*P* < 0.001) [17]. The possible reason for this is that the NASH stage involves inflammation and hepatocyte damage, in contrast to simple fatty liver, which includes inflammatory cell infiltration. As NASH progresses, it leads to hepatic fibrosis, an excessive deposition of collagen fibers in the liver tissue. This fibrosis, which occurs as a response to chronic liver injury, can impair hepatic function and even progress to cirrhosis, resulting in the hardening of liver tissue. Consequently, the hepatic Young’s modulus value increases, which may explain the contrary results observed in this study. However, the study did not find an association between STE and STQ measurements in the right lobe of the liver and fatty liver. This may be due to the fact that children in this age group generally have a higher basal metabolic rate than adults, and the fat deposition in the liver occurs over a relatively short period, which may not cause substantial changes in the liver. As a result, the sensitivity of STE and STQ to detect mild fatty liver degeneration is lower, particularly in children with fatty liver who have normal liver function indicators. Alternatively, the right lobe of the liver was larger in size, and fat was unevenly deposited in the right lobe of the liver, and only some of the liver tissues were affected by sampling errors, resulting in a statistically non-significant difference in Young's modulus values between the two groups.

In the present study, we found that in the livers of normal children, Young's modulus measurements in the left lobe were higher than those in the right lobe when measured using the STE mode (Table **3**). We hypothesize that the difference in stiffness values between the left and right lobes may be attributed to variations in anatomical structure, blood vessel distribution, and hepatocyte density. In addition, the left lobe of the liver was not covered by ribs, and the probe was placed directly on top of the liver, so the operator's pressure was not uniformly controlled, and the left lobe of the liver, which was adjacent to the large blood vessels and the heart, was subject to the influence of the blood flow rate and the heartbeat. Taken together, these two reasons led to higher values of Young's modulus measured in the left lobe of the liver than in the right lobe. Fontanilla T reported that AFRI measured higher stiffness values in the left lobe of the liver than in the right lobe, with greater stability of the measurements in the left lobe [18]. These findings are consistent with those observed in our study. However, in fatty liver, the difference between the left and right lobes of the liver was not statistically significant in the two elastography modes, STE and STQ. Although there are differences in the anatomical structure, vascular distribution, and hepatocyte density between the right and left lobes of the liver, the variability in stiffness between the right and left lobes is reduced or eliminated as the liver tissue becomes more homogeneous as a whole due to the effects of fat deposition. In addition, fatty liver may also lead to changes in the stiffness of the liver, which is consistent with the results of the current study. This could explain the more consistent stiffness values observed between the right and left lobes of the liver. This concordance may have resulted in liver stiffness values that were not statistically significant between the right and left lobes of the liver measured for steatohepatitis.

This study conducted a multivariate logistic analysis to explore the independent risk factors of NAFLD. The results showed that BMI and the Young's modulus value of the left lobe of the liver were independent risk factors for NAFLD. The OR values were 2.61 (95% CI: 2.01 - 3.41) and 0.61 (95% CI: 0.41 - 0.90), respectively (Table **4**). This may be attributed to the fact that a higher BMI in NAFLD patients correlates with greater lipid accumulation in the liver, leading to more severe hepatic steatosis. In addition, the Young's modulus value of the left lobe of the liver was significantly negatively correlated with the prevalence of fatty liver. Clinically, children with a lower Young's modulus value in the left lobe of the liver should be considered at higher risk for NAFLD, and a comprehensive assessment should be conducted based on their clinical indicators. Moreover, this study only found a correlation between the Young's modulus value of the left lobe of the liver and NAFLD. No association was found between the Young's modulus values of the right lobe of the liver, STQ-derived Young's modulus value, and the Young's modulus value of the left lobe of the liver and the occurrence of NAFLD. The occurrence of fatty liver is a complex process involving multiple factors. In addition to liver stiffness, factors, such as lifestyle, genetic background, nutritional status, and metabolic syndrome, may also play a role. The Young's modulus value may, therefore, be just one indicator among many. In the future, we aim to enhance the dataset by including additional potential influencing factors to better understand the pathogenesis and determinants of NAFLD.

**Fig. (1):**
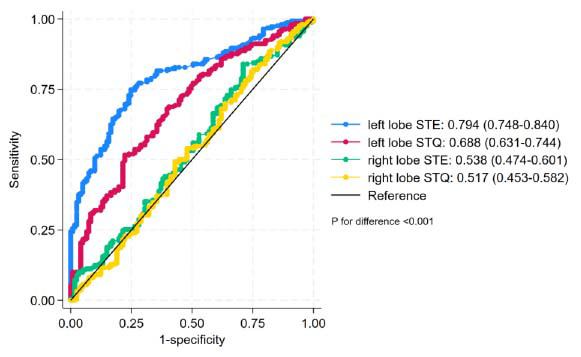
ROC curve of fatty liver.

**Fig. (2):**
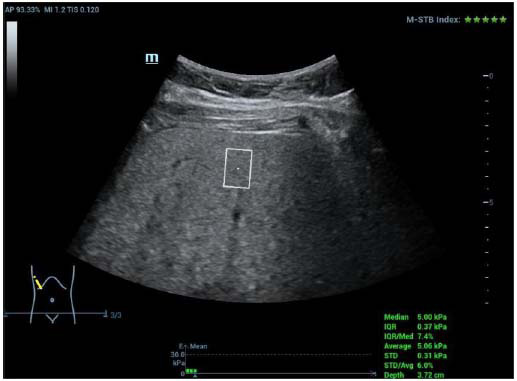
Measurement by STQ in the left lobe of children with NAFLD.

**Fig. (3):**
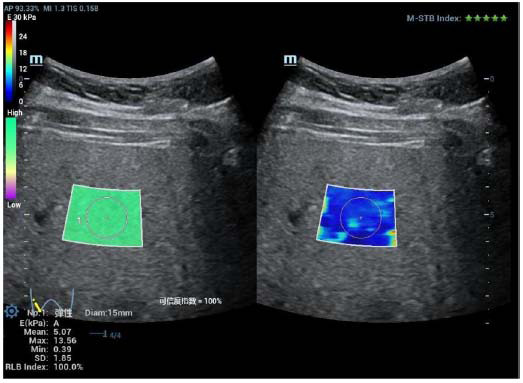
Measurement by STE in the left lobe of children with NAFLD.

**Fig. (4):**
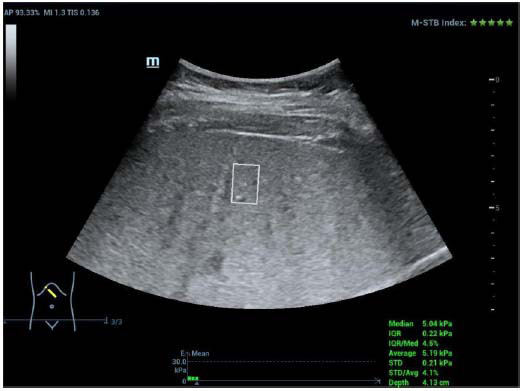
Measurement by STQ in the right lobe of children with NAFLD.

**Fig. (5):**
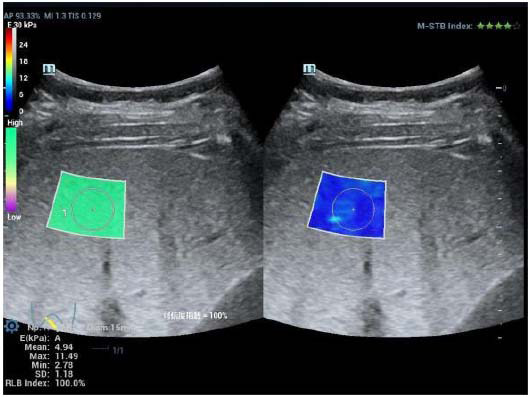
Measurement by STE in the right lobe of children with NAFLD.

A total of 372 children were divided into a fatty liver group and a non-fatty liver group. The optimal diagnostic cut-off value for STE to diagnose steatohepatitis in the left lobe of the liver was 5.890 kPa, with a diagnostic sensitivity of 74.8%, a specificity of 75.2%, and an area under the ROC curve (AUC) of 0.794. The optimal diagnostic cut-off value for STQ to diagnose fatty liver in the left lobe of the liver was 8.050 kPa, with a diagnostic sensitivity of 51.6%, a specificity of 77.7%, and an area under the ROC curve (AUC) of 0.688 (Table **5** and Figs. **1**-**5**). The analysis of area under the ROC curve (AUC) revealed that the Young's modulus value obtained from SWE of the liver’s left lobe indicated high sensitivity and specificity, highlighting strong diagnostic performance. Compared with Young's modulus value derived from STQ of the left lobe of the liver, STE technology demonstrated superior diagnostic accuracy (specific AUC value), providing a more valuable quantitative basis for clinical diagnosis. Therefore, this study recommends the use of the Young’s modulus value obtained from SWE of the liver’s left lobe as the preferred diagnostic parameter. The findings of studies by He [19] and Garcovich *et al.* [16] demonstrated higher sensitivity and specificity compared to the present study. This discrepancy may be attributed to the age distribution within the fatty liver group, as well as the substantial variation in the number of children across different age groups in the sample, potentially introducing statistical bias favoring the age group with a larger representation. It may also be related to variations in liver function among the children in the fatty liver group, as both normal and abnormal liver function cases were included. Due to the limited number of children with abnormal liver function, a detailed subgroup analysis was not performed. These factors, age distribution and liver function variability, may have influenced the sensitivity and specificity observed in the present study and should be carefully addressed in future research to enhance the robustness of the findings.

The limitations of this study are as follows:(1) This study is cross-sectional in design, which limits its ability to establish causal relationships. To further investigate the association between contributing factors and the development of fatty liver, prospective studies will be conducted in the future. (2) The children examined in this study had a wide range of ages, and in the future, children of different ages will be meticulously stratified to obtain precise cut-off values. (3) In this study, the ROC curve was employed to identify the optimal cut-off value for the liver elasticity index; however, the association between this threshold and the presence of fatty liver did not reach statistical significance. Future studies with larger sample sizes are needed to establish reliable reference ranges, thereby providing a stronger theoretical foundation for the diagnosis of pediatric fatty liver disease. (4) Due to limitations in the available data, the extent of hepatic fat accumulation in children diagnosed with fatty liver was not graded, preventing an assessment of the relationship between Young’s modulus values and varying degrees of steatosis. Future studies will incorporate data on fat accumulation severity to enable a more detailed analysis of this relationship. (5) Although this study adjusted for gender, age, and body mass index, potential confounding factors remain unaccounted for due to limitations in the available data. Additional data collection and analysis will be necessary to control for these variables in future research.

## CONCLUSION

In conclusion, this study found that liver elasticity indices of STE and STQ were lower in the left lobe of the liver in children with fatty liver compared to children without fatty liver.It provides a certain theoretical basis for choosing appropriate indexes for clinical diagnosis of fatty liver in children using ultrasound, but the results of multifactorial analysis have not yet found an association between liver elasticity indexes based on STE and STQ shear wave elastography measurements and fatty liver in children, and further research is still needed.

## Figures and Tables

**Table 1:** Baseline information of the non-fatty liver group and the fatty liver group.

-	Non-fatty Liver Group (n = 251)	Fatty Liver Group (n = 121)	*P*-value
Age^a^, years	6.59 ± 2.77	10.23 ± 2.21	<0.01
Sex, male (%)	129(51.39)	88 (72.73)	<0.01
Height^a^, m	1.26 ± 0.20	1.53 ± 0.16	<0.01
Weight^a^, kg	28.55 ± 12.66	64.00 ± 17.59	<0.01
BMI^a^, kg/m^2^	16.89 ± 2.93	26.27 ± 3.60	<0.01
BSA^a^, m^2^	1.01 ± 0.30	1.64 ± 0.29	<0.01

BMI, body mass index; BSA, body surface area. a: Mean ± standard deviation.

**Table 2:** Comparison of Young's modulus values between non-fatty and fatty liver groups.

-		Non-fatty Liver Group (n = 251)		Fatty Liver Group (n = 121)		*P*
STE, kPa						
Right lobe of the liver		6.33 ± 1.42		6.12 ± 1.34		0.182
Left lobe of the liver		7.31 ± 2.09		5.47 ± 1.06		<0.05
STQ, kPa						
Right lobe of the liver		7.51 ± 2.10		7.47 ± 2.44		0.881
Left lobe of the liver		8.84 ± 3.26		7.01 ± 2.15		<0.05

**Abrreviations:**STE: Sound touch elastography; STQ: Sound touch quantification.

**Table 3:** Comparison of mean values of the Young's modulus of the left and right lobes of the liver.

-	Left Lobe of the Liver	Right Lobe of the Liver	*P*
Non-fatty liver group			
STE	7.31 ± 2.09	6.33 ± 1.42	<0.05
STQ	8.78 ± 3.22	7.51 ± 2.12	<0.05
Fatty liver group			
STE	5.47 ± 1.06	6.12 ± 1.34	<0.05
STQ	7.01 ± 2.15	7.47 ± 2.44	0.117

**Abbreviations:** STE: Sound touch elastography; STQ: Sound touch quantification.

**Table 4:** Multivariate logistic regression analysis of fatty liver.

-	β	S.E	Z	*P*	OR (95%CI)
BMI	0.96	0.13	7.12	<0.001	2.61 (2.01-3.41)
Left lobe of liver STE	-0.50	0.20	-2.50	0.012	0.61 (0.41-0.90)

**Abbreviations:**BMI, body mass index; STE: Sound touch elastography.

**Table 5:** ROC curve analysis of STE and STQ in the right and left lobes of the liver.

-	AUC	Sensitivity	Specificity	Cut-off Value
STE in the left lobe of the liver	0.794	0.748	0.752	5.890
STQ in the left lobe of the liver	0.688	0.516	0.777	8.050
STE in the right lobe of the liver	0.538	0.840	0.289	5.050
STQ in the right lobe of the liver	0.517	0.812	0.256	5.780

STE: Sound touch elastography; STQ: Sound touch quantification.

## Data Availability

The research data are confidential due to ethical restrictions.

## LIST OF ABBREVIATIONS

STQ: Sound touch quantification
STE: Sound touch elastography
NAFLD: Non-alcoholic fatty liver disease
NAFL: Non-alcoholic fatty liver
NASH: Non-alcoholic steatohepatitis
UE: Ultrasound elastography

## STANDARDS OF REPORTING

STROBE guidelines were followed.
